# Addition of collagen type I in agarose created a dose-dependent effect on matrix production in engineered cartilage

**DOI:** 10.1093/rb/rbac048

**Published:** 2022-08-11

**Authors:** Gabriel R López-Marcial, Keerthana Elango, Grace D O’Connell

**Affiliations:** Department of Mechanical Engineering, University of California-Berkeley, 6141 Etcheverry Hall, Berkeley CA 94720, USA; Department of Mechanical Engineering, University of California-Berkeley, 6141 Etcheverry Hall, Berkeley CA 94720, USA; Department of Mechanical Engineering, University of California-Berkeley, 6141 Etcheverry Hall, Berkeley CA 94720, USA; Department of Orthopaedic Surgery, University of California-San Francisco, 500 Parnassus Ave MU-320W, San Francisco CA 94143, USA

**Keywords:** hydrogel, tissue engineering, composition, agarose-collagen matrix, mechanics

## Abstract

Extracellular-matrix composition impacts mechanical performance in native and engineered tissues. Previous studies showed collagen type I-agarose blends increased cell-matrix interactions and extracellular matrix production. However, long-term impacts on protein production and mechanical properties of engineered cartilage are unknown. Our objective was to characterize the effect of collagen type I on the matrix production of chondrocytes embedded in agarose hydrogels. We hypothesized that the addition of collagen would improve long-term mechanical properties and matrix production (e.g. collagen and glycosaminoglycans) through increased bioactivity. Agarose hydrogels (2% w/v) were mixed with varying concentrations of collagen type I (0, 2 and 5 mg/ml). Juvenile bovine chondrocytes were added to the hydrogels to assess matrix production over 4 weeks through biochemical assays, and mechanical properties were assessed through unconfined compression. We observed a dose-dependent effect on cell bioactivity, where 2 mg/ml of collagen improved bioactivity, but 5 mg/ml had a negative impact on bioactivity. This resulted in a higher modulus for scaffolds supplemented with lower collagen concentration as compared to the higher collagen concentration, but not when compared to the control. In conclusion, the addition of collagen to agarose constructs provided a dose-dependent impact on improving glycosaminoglycan production but did not improve collagen production or compressive mechanics.

## Graphical Abstract

**Figure:**
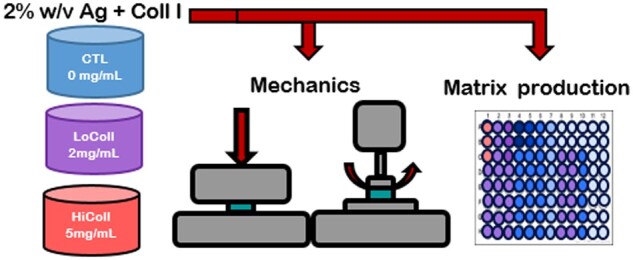

## Introduction

Gradients in tissue composition are found throughout the body, connecting materials with different stiffnesses, in turn, creating gradients in mechanical properties [1]. Mechanical gradients are of particular importance at interfaces of soft and hard tissues, like tendon or cartilage to bone, where sudden mismatches in stiffness create stress concentrations that contribute to tissue failure [2]. Gradients in fiber architecture and tissue composition can alleviate stress concentrations between materials with mismatched mechanical properties [1, 3]. Replicating tissue and mechanical gradients is important for the successful integration of engineered tissue with surrounding native tissues.

Gradients in tissue stiffness, fiber orientation and tissue composition exist through the thickness of articular cartilage [4]. Cartilage is commonly described as having three zones whose properties are directly affected by composition: the stiff superficial zone, where collagen type II is organized parallel to the surface, a transition or mid-zone with high quantities of glycosaminoglycans (GAG) and randomly aligned collagen fibers, and a deep zone near the bone where collagen is oriented perpendicular to the surface [4, 5]. This organization results in increasing compressive and shear moduli from the superficial to deep zone, while tensile modulus decreases, allowing energy to be dissipated through the depth of the tissue [2, 6].

Agarose hydrogels have been specifically used for cartilage tissue engineering because of their ability to maintain a rounded chondrogenic phenotype, which has been observed to stimulate GAG production, resulting in compressive mechanical properties that approach native values [7]. Collagen type I hydrogels mixed with other gels, including agarose, alginate and collagen type II, have resulted in increased cell-matrix interactions and GAG production [8–10].

Studies that used an agarose-collagen mixture have shown an increase in cell bioactivity. These studies have evaluated gene expression over multiple weeks [9, 11, 12]. However, we have little insight into whether collagen-agarose blends develop functional engineered cartilage, as gene expression is an imperfect indicator of protein production [13, 14]. Hydrogels by themselves exhibit relatively weak mechanical properties and are dependent on extracellular-matrix deposition to approach native mechanical properties. While the addition of collagen may result in weaker initial mechanical properties, due to a disruption in the agarose network [9], potential increases in protein production due to greater bioactivity may result in greater long-term benefits. Thus, the impact of collagen on *de novo* matrix production and mechanics within an agarose-based system may influence biomimetic hydrogel scaffold designs.

Thus, the objective of this work was to characterize the effect of collagen type I on the matrix production of bovine chondrocytes embedded in an agarose scaffold. We hypothesized that the addition of collagen type I will increase extra-cellular matrix production and mechanical properties. Furthermore, a low and high dose of collagen was assessed to determine whether there is a dose-dependent effect on matrix production and mechanical behavior.

## Materials and methods

### Sample preparation

An agarose gel stock was created by dissolving type VII agarose powder (Sigma-Aldrich, St Louis, MO) in 0.15M phosphate-buffered saline (1× PBS) at a concentration of 6% weight over volume (w/v; 1 g/100 ml) by heating to 121°C in an autoclave for ∼20 min. The stock gel was then diluted with saline and type I collagen (Bovine, Advanced Biomatrix, CA) to obtain a final concentration of 2% agarose gels. Collagen was added at concentrations of 0 mg/ml for the control (CTL), 2 mg/ml for the low concentration group (LoColl) or 5 mg/ml for the high collagen group (HiColl). Collagen concentrations were chosen to facilitate comparisons to previous literature on agarose-collagen gels [9]. For cell-based experiments, the stock gel was cooled to ∼40°C before adding chondrocytes (final concentration of 30 × 10^6^ cells/ml) and casting the mixture between glass slides. The gel was allowed to cool to room temperature before using a biopsy punch to obtain cylindrical samples (4 mm diameter).

### Chondrocyte isolation and culture

Junior bovine knee joints were obtained from an abattoir (Green Village Packing, NJ), and chondrocytes were obtained by digesting cartilage with type 4 collagenase (activity 375 units/mg dry weight; Worthington Biochemical, Lakewoond, NJ) dissolved in media (DMEM with 5% FBS, 1% non-essential amino acids, 1% TES, 1% BES, 1% HEPES, 1% sodium bicarbonate, 1% penicillin-streptomycin antimycotic (PS/AM), 2% minimum essential aminoacids) and shaken overnight inside an incubator (37°C, 5% CO_2_). Cells (1 × 10^6^ per vial) were frozen in liquid nitrogen with DMSO until use. Frozen vials were thawed rapidly (<2 min) in a 37°C water bath and then plated in a culture flask. Cells were cultured in growth media (DMEM with 10% FBS, 1% PS/AM, 5 ng/ml FGF, 10 ng/ml PDGF and 1 ng/ml TGFβ-1) until passage 6 until they were seeded into gels.

### Rheometry

Oscillatory rheometry (Anton Paar) was performed on cylindrical, acellular gel samples (height = 2.3 mm; *n* = 8) using a sandblasted parallel plate with a diameter of 8 mm and a gap size of 2 mm. Samples were created with a diameter of 8 mm to match the diameter of the testing plate. A temperature ramp (2°C/min) was performed from 25 to 37°C, followed by a 5-min isothermal step at 37°C with an oscillatory shear of 1% at 1 Hz. Storage (G’) and loss (G”) moduli were defined as the average of all values recorded at the isothermal step (50 data points).

### Engineered cartilage

#### Construct culture

Cylindrical samples (diameter = 4 mm, thickness = 2.3 mm; *n* = 5 per group for each timepoint for mechanics and biochemical assays) were cultured in serum-free chondrogenic media (DMEM with 4.5 g/l glucose and l-glutamine, 1% ITS+Premix, 1% penicillin-streptomycin, 100 μg/ml sodium pyruvate) for 4 weeks. Media was changed three times a week and supplemented to final concentrations of 50 μg/ml ascorbic acid and 100 nM dexamethasone on the day of feeding. Media was additionally supplemented with 10 ng/ml of TGFβ-3 for the first 2 weeks of culture.

#### Compressive mechanics

Stress-relaxation tests were performed to 10% strain to evaluate compressive Young’s modulus and time-dependent behavior (10% strain, rate = 2%/min) on cell-laden cylindrical samples (diameter = 4 mm, thickness = 2.3 mm) on Days 1 and 2 (Week 0) and Days 29 and 30 (Week 4) under unconfined compression in a saline bath. Sample diameter and height for each individual sample were measured with a caliper prior to testing. Time points are labeled as weeks instead of days for convenience, as groups were tested within a 48-h period. Young’s modulus was calculated as the slope of the linear portion of the loading curve during the ramp to 10% strain. Relaxation was defined as the stress after 30 min divided by the peak stress at the end of the ramp subtracted from 1.0 and presented a percentage. Therefore, 0% relaxation represents a fully elastic material while 100% relaxation represents a material that has undergone complete relaxation.

#### Cell viability and imaging

Samples were stained and imaged in Weeks 1 and 3 to assess cell viability and observe changes in morphology (*n* = 3 from each group; Live/Dead kit, Life Technologies, Carlsbad, CA). The staining solution contained 0.5 μl/ml of Calcein AM to stain live cells and 2 μl/ml of ethidium homodimer-1 in 1× PBS. Samples were protected from light and submerged in staining solution for 20 min at room temperature, rinsed with 1× PBS, then imaged within 2 h. Z-stack images were collected at 533 nm for live cells (green) and 640 nm for dead cells (red) using a confocal microscope (Praire Technologies; 10× objective). A custom MATLAB program was used to estimate the number of objects in each channel using a grayscale threshold (grayscale level > 35 000). Cell viability was measured as the number of live cells divided by the total cell count in the image stack and reported as a percent.

#### Biochemical content

After mechanical testing, samples were re-hydrated (>20 min), weighed, and collected to measure DNA, GAG and collagen contents. Specimens were lyophilized (Labconco, Kansas City, MO) for 48 h to determine the dry weight and digested overnight at 56°C with Protenaise K (MP Biomedical, Burlingame, CA). DNA content was determined using the fluorescent PicoGreen assay, GAG content was determined using the colorimetric dimethyl methylene blue assay, and collagen content was determined using the hydroxiproline (OHP) assay. Both GAG and OHP content were normalized by DNA content and the sample wet weight.

### Statistics

Due to the relatively small sample size, normality was not assumed. A one-way non-parametric ANOVA (Kruskal–Wallis) was performed to assess differences among groups. Comparisons were evaluated for initial and final properties (i.e. in Weeks 0 and 4). A Dunn’s multiple comparison *post hoc* was used to determine specific *P*-values between groups. Significance was assumed for *P* ≤ 0.05. Statistical analyses were performed using GraphPad PRISM version 9.3 (GraphPad, San Diego, CA).

## Results

### Changes in mechanical properties

**Figure 1.**
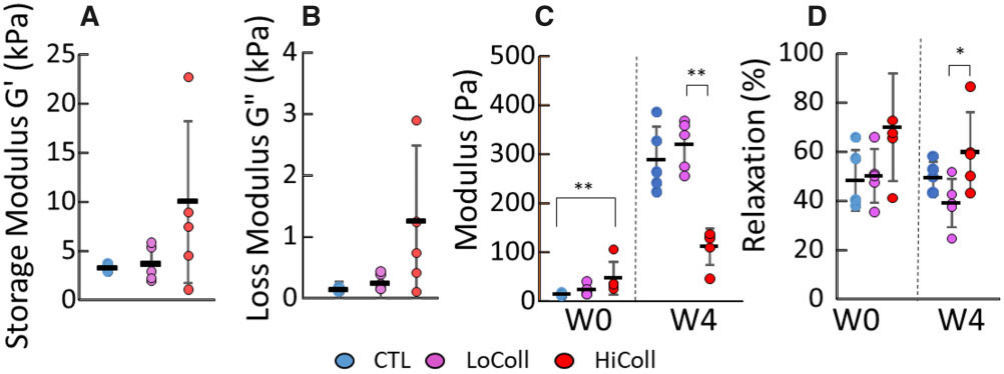
(**A,B**) Rheological properties of acellular hydrogels. (A) Storage modulus and (B) loss modulus at 37°C. No significant differences were observed between groups (*P* > 0.2). (**C,D**) Initial (W0) and final (W4) compressive mechanics of engineered cartilage constructs. (C) Linear region young’s modulus and (D) percent relaxation measured during the hold period of a stress-relaxation test. Each sample is plotted as a data point, with rectangles and bars representing mean ± standard deviation. * represents *P* < 0.05. ** represents *P* < 0.0.1.

Acellular mechanical properties were evaluated using a rheometer*.* The storage modulus for the control group was G’ = 3.6 ± 1.8 kPa and the loss modulus was G” = 0.2 ± 0.1 kPa. There were no significant differences observed in storage or loss modulus with the addition of collagen to the hydrogel (*P* = 0.2 for G’; *P* = 0.06 for G”; Fig. 1). Variability in measured mechanical properties increased with the addition of collagen, which impacted the power of the analysis (*β* = 0.14). There were significant differences in the initial mechanical properties of seeded scaffolds tested under unconfined compression (*P* < 0.01; Fig. 1C—Week 0). Specifically, the Young’s modulus of the HiColl group was 47.8 ± 33.0 kPa, which was more than 3× greater than the CTL group (14.4 ± 2.6 kPa) at Week 0 (Fig. 1C; *P* = 0.009). All scaffolds experienced 50–60% relaxation during the 30-min hold, with no significant differences with respect to collagen supplementation (*P* = 0.11; Fig. 1D). Final bulk mechanical properties measured at Week 4 showed that the Young’s modulus for CTL and LoColl groups were 2.6× stiffer than the HiColl group (*P* < 0.01; Fig. 1C). There were no significant differences in compressive Young’s modulus between CTL and LoColl groups. At Week 4, the percent of relaxation for the LoColl group was 21% lower than HiColl (38.9 ± 9.8% and 59.7 ± 16.5%, respectively; *P* = 0.02; Fig. 1D. However, there were no significant differences in percent relaxation with respect to the CTL group (*P* = 0.35).

### Changes in construct size

**Figure 2.**
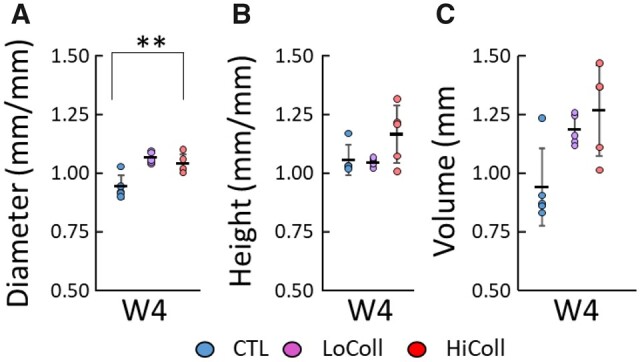
Dimensions of engineered cartilage constructs at Week 4 (W4), normalized by dimensions at Week 0: (**A**) diameter (d), (**B**) height (h) and (**C**) volume ($V=\pi {\left(\frac{d}{2}\right)}^{2}h$). Each sample is plotted as a data point, with rectangles and bars representing mean ± standard deviation. ** represents *P* < 0.01.

The HiColl group presented a larger diameter than the CTL group when normalized by Week 0 diameter (*P* = 0.006; Fig. 2A). This difference was due to a 6% decrease in diameter for the CTL group and a ∼10% increase in diameter for the HiColl group from initial values (i.e. Week 0). However, these differences did not translate to significant differences in construct height or volume at Week 4 (*P* > 0.05; Fig. 2B, C).

### Changes in matrix production

**Figure 3.**
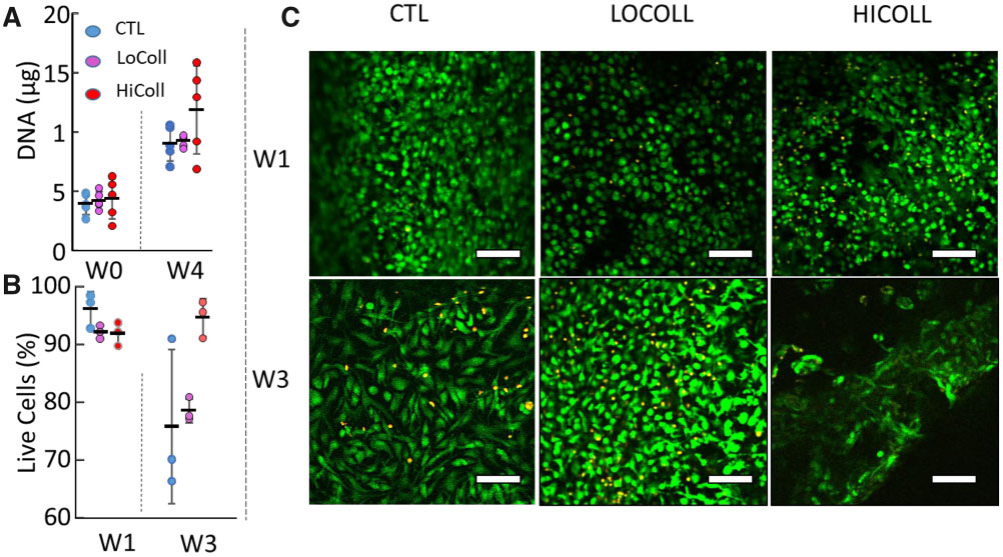
(**A**) DNA content, (**B**) percentage of living cells and (**C**) live/dead imaging, where green represents live cells and yellow represents dead cells (Full color image may be found in the online version of this article). Scale bars = 100 μm. No statistical differences were observed for DNA content or percentage of living cells counted at either time point (*P* > 0.4). Each sample is plotted as a data point, with rectangles and bars representing mean ± standard deviation.

No differences in DNA content were observed between groups at Week 0 or Week 4, suggesting that cells were seeded evenly, and the added initial collagen did not alter cell proliferation (Fig. 3A). At Week 1, all groups had high cell viability (>90%; *P* = 0.23; Fig. 3B), and there were no noticeable differences in cell morphology (Fig. 3C—top row). After 3 weeks of culture, cell viability was lower for the CTL (75.8 ± 13.2%) and LoColl groups (78.6 ± 2.1%; Fig. 3B), and cells from all groups showed an elongated phenotype (Fig. 3C—bottom row). Dedifferentiation of chondrocytes was observed in all gels at Week 3 (Fig. 3C), which may be due to increases in substrate stiffness with matrix deposition or to using passaged cells rather than primary cells [15–17].

**Figure 4.**
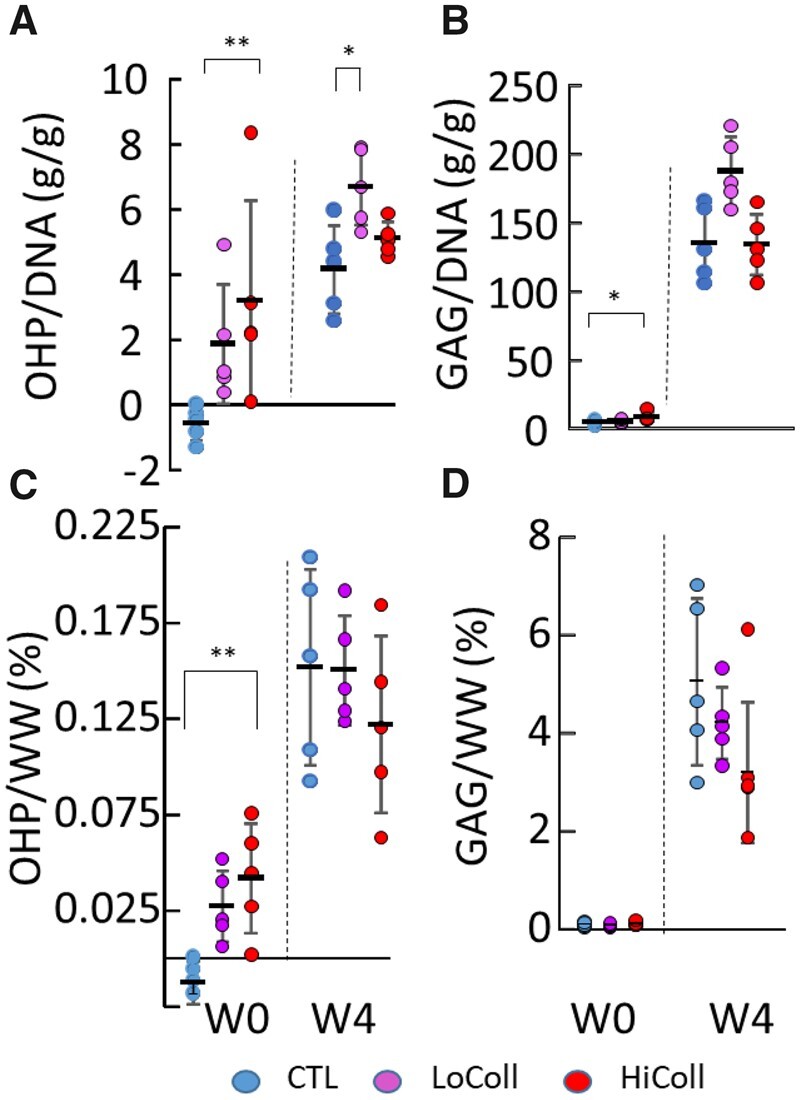
Biochemical content was normalized by (**A**, **B**) DNA content to assess biosynthesis and (**C**, **D**) wet weight (WW) to compare to data in the literature. (A, C) * represents *P* < 0.05. Each sample is plotted as a data point, with rectangles and bars representing mean ± standard deviation. ** represents *P* < 0.01.

The addition of collagen resulted in statistically significant differences in the initial collagen content, as expected (Fig. 4A, C—Week 0). In Week 4, the LoColl group was more biologically active than CTL, based on OHP/DNA (LoColl = 6.7 g/g CTL = 4.2 g/g; *P* = 0.02), but not significantly more active than the HiColl group (5.1 g/g; *P* = 0.23, Fig. 4A). There was also a trend for greater matrix biosynthesis in the LoColl group with respect to GAG production (Fig. 4B; *P* = 0.059). However, these differences in matrix biosynthesis were not reflected in overall differences in tissue composition when normalized by wet weight, as commonly performed in the literature (Fig. 4C, D).

## Discussion

The objective of this study was to evaluate the effect of incorporating collagen within agarose to increase matrix synthesis during a month-long 3D culture. GAG growth in CTL and LoColl groups were similar to previously reported values for agarose-only gels [7], resulting in compressive properties within the range of native values (240–850 kPa) [18]. A previous study found that the addition of collagen type I in the range of 2–4.5 mg/ml increased GAG production from nucleus pulposus cells embedded in agarose [9]. The addition of collagen to the agarose hydrogel in this study demonstrated a dose-dependent effect on cell bioactivity, where 2 mg/ml of collagen improved bioactivity but the higher concentration had a negative impact on cell bioactivity.

The addition of collagen to the agarose scaffold only altered elastic mechanical properties at higher strains. The increase in Young’s modulus of agarose-collagen gels agreed with an experimental study by Kopf *et al*. [12]. The increase in stiffness may be due to greater fiber engagement as the non-fibrillar agarose matrix experiences deformation [19]. Similarly, a lack of observed changes with increased collagen on rheological mechanics agreed with data from Cambria *et al.*, when correcting the reported *P*-values for the number of observations (i.e. running a one-way ANOVA rather than multiple *t*-tests) [9]. Discrepancies in absolute values between data reported in this study and Cambria *et al*. [9] may be due to differences in surface treatment and geometry of the parallel plates used in rheometry [20]. The discrepancy in findings between testing modalities may be explained by a decrease in fiber engagement during rheometry. That is, unconfined compression testing was performed at 10% strain, while oscillatory rheometry reached maximum strains of 1%, which is not high enough to detect strain-stiffening behavior in collagen gels [21]. The non-linear behavior of engineered cartilage suggests that greater differences may have been observed at higher strains [22]; however, 10% strain was used in this study to represent moderate physiological loading and compare to existing data in the literature [23–25]. Initial time-dependent relaxation behavior was not altered by collagen, agreeing with previous work that the evaluated time-dependent behavior of agarose-collagen gels with indentation testing [26]. The relaxation behavior of the agarose-collagen blend is thought to be dominated by the agarose matrix [26, 27]; thus, it was unaffected by the relatively low quantities of collagen added (0.1–0.25% of total w/v).

A dose-dependent effect was also observed in matrix deposition, with the greatest GAG/DNA and OHP/DNA content at Week 4 in the LoColl group (Fig. 4A, B). GAG content is directly linked to compressive mechanics, which was also observed in this study, where the HiColl group was initially stiffer than the CTL and LoColl groups (Week 0) but was less stiff at Week 4 when the GAG content was also lower. The lower GAG content also resulted in a greater relaxation response when compared to other groups (Fig. 1D), agreeing with trends of energy dissipation seen in native articular cartilage [28]. Extra-cellular matrix deposition did not affect overall construct size between groups, as all groups showed similar values for volume at Week 4 relative to their initial volume (Fig. 2C). Previous research has shown that collagen gels may experience a decrease in size over time due to contractile cell forces on fibers [9, 29], but this was not observed in this study. The lack of scaffold contraction was likely due to the much higher agarose concentration and the use of chondrocytes, both of which have been shown to counter the effects of cell-mediated contraction in collagen gels [11, 30].

While collagen type II is the predominate type in native cartilage, collagen type I was used here based on previous *in vitro* studies [11, 31]. However, a recent study showed that the blending both type I and II collagen had a greater impact on GAG production and gene expression (Sox9, aggrecan, Coll I, II, X) when compared to pure collagen I scaffolds, suggesting the mechanical function of our gels could have been further improved by including both collagen type I and II [10]. Improving collagen production in engineered cartilage has been a significant challenge. Degrading the early production of GAGs with chondroitinase ABC has resulted in greater improvements in collagen production (75% increase over wet weight when compared to the control) than what was observed in this study [17].

This study is not without limitations. First, collagen is expected to have a greater impact on tensile properties, which was not assessed here due to the challenges of culturing longer specimens for tensile testing. Second, this study had a low sample size per group due to time constraints on mechanical testing and the living nature of the material. This low sample size made it difficult to infer statistical meaning in findings such as GAG/WW, where a power analysis suggests an additional 15 samples would have detected differences between groups. Thirdly, a Western blot would provide deeper insight into the type of collagen being produced [32, 33]. We did not investigate the specific collagen types in this study due to the relatively minimal benefits observed in supplementing the agarose scaffold with collagen under static culture conditions. Regardless, our findings support the notion that collagen supplementation at a lower concentration can increase chondrocyte bioactivity within an agarose hydrogel, as suggested in the literature. However, the increase in bioactivity did not greatly increase collagen production outside the range of previously reported values for engineered cartilage (∼1.5%/WW versus native ∼10%/WW). Thus, the overall impact of using a collagen-agarose blend to increase collagen production in engineered cartilage is low under static culture conditions.
